# Pillar Zero: a temporal anchor for patient-centered Patient Blood Management (PBM)

**DOI:** 10.1016/j.bjane.2026.844801

**Published:** 2026-07-22

**Authors:** Flávio Takaoka, Maria José Carvalho Carmona, Renato Carneiro de Freitas Chaves, Paulo Roberto Silva Garcez dos Santos

**Affiliations:** a Hospital Israelita Albert Einstein, São Paulo, SP, Brazil; b Faculdade de Medicina da Universidade de São Paulo (FMUSP), Departamento de Anestesiologia, São Paulo, SP, Brazil; c Massachusetts Institute of Technology, Cambridge, MA, United States

## Introduction

The three pillars of Patient Blood Management (PBM) ‒ detection and management of anemia, minimization of blood loss, and optimization of physiological tolerance ‒ represent a well-validated, guideline-endorsed framework for reducing perioperative transfusion, complications, and mortality.^1^, ^2^, ^3^ The clinical rationale for each pillar is well established, yet implementation has lagged far behind what the evidence warrants. The reasons are well characterized and include coordination failures, over-reliance on transfusion as residual solution, and cultural inertia.^4^ This editorial argues that one specific failure underlies all others: *the absence of a temporal activation point* ‒ a failure that is, at its root, a failure of patient-centered pathway design.

## Evidence of a missed opportunity

Two recent Brazilian cohort studies define the scope of this failure. A retrospective study from a quaternary public hospital found that 42.1% of surgical patients were anemic preoperatively ‒ independently associated with 30-day mortality (RR = 1.69, 95% CI 1.44–1.99), transfusion (RR = 4.44, 95% CI 3.90–5.06), and ICU admission (RR = 1.09, 95% CI 1.04–1.16).^5^ Over five consecutive years, with no policy changes, the burden remained unchanged; only 10.5% of patients with moderate anemia underwent etiological investigation.^5^ A second cohort of 23,579 adults undergoing elective noncardiac surgery documented a severity-dependent mortality gradient: mild anemia doubled in-hospital mortality (adjusted RR = 2.5), moderate anemia increased it more than fourfold (RR = 4.6), and severe anemia carried a nearly 25-fold increase (RR = 24.7, 95% CI 13.3–46.0).^6^ The median interval between hemoglobin measurement and surgery was 26.7 days ‒ below the threshold for oral iron but within the window for intravenous iron ‒ yet no intervention was initiated. This window is sufficient for intravenous iron; to preserve the option of oral iron, activation must occur even earlier, at surgical indication.^6^ Internationally, the CPOC Anemia Guideline (2025) reports that 67% of anemic surgical patients received no preoperative treatment, including 31% with severe anemia.^7^ These figures do not represent a failure of the three pillars; they point to a problem upstream.

## The structural problem: provider-centered pathways and the collapsed time window

Perioperative pathways are organized around provider workflow rather than patient needs.^8^^,^^9^ The Full Blood Count (FBC) is ordered at the preoperative assessment ‒ set for scheduling convenience, typically days before surgery. By the time hemoglobin is communicated, the surgical date is imminent and the clinical decision binary: proceed with an anemic patient, or cancel. Neither option deploys a PBM pillar; both bypass the patient as an agent in their own preparation. This is not a failure of education or protocol adherence. It is a structural failure to anticipate patient needs.

Even when the FBC is ordered in advance, results routinely fall between specialists: the surgeon holds no mandate to act on them, the anaesthetist was not notified, and no named agent owns the finding. The timing failure and the ownership gap are inseparable.^4^^,^^8^ Oral iron requires a minimum of four weeks to produce a clinically meaningful hemoglobin increment;^7^ intravenous iron requires 2–3 weeks.^7^^,^^10^ The stable anemia prevalence documented over five years in Brazilian surgical populations reflects a system in equilibrium with untreated preoperative anemia ‒ an equilibrium the three pillars alone cannot break because they activate after the therapeutic window, and the patient has already been failed.^5^

The timing failure is compounded by an ownership gap: preoperative anemia is nobody’s explicit institutional responsibility. The 2025 ESAIC guidelines on preoperative assessment explicitly recognize this gap: even after a full preoperative workup, the assessment must be updated within 48 hours of surgery to allow rescheduling when optimization ‒ including correction of preoperative anaemia ‒ remains incomplete.^11^ Yet within this framework, if anemia detection occurs only at the preoperative assessment, the remaining time window may be insufficient for oral iron therapy. The deeper obstacle is cultural ‒ the dominant assumption that anemia is the patient’s condition, the waiting list an administrative problem, and transfusion the residual solution. Making inaction visible and timestamped is the first step toward contesting them.

## Pillar Zero: definition and operational framework

Pillar Zero is defined as the mandatory activation of the PBM workflow at the time surgery is indicated or scheduled, governed by explicit, computationally verifiable patient-level criteria. Two guideline frameworks define its operational basis. The NICE NG45 guideline establishes that FBC is indicated for patients undergoing major surgery (all ASA grades) and for patients undergoing intermediate surgery classified as ASA III or IV.^12^ It addresses both structural failures simultaneously: the activation event ties the FBC to a specific, documentable moment week to months before surgery, and explicit ownership is assigned to the surgical team at that same consultation, so that the result does not fall between specialists. Pillar Zero does not directly challenge the cultural beliefs described above, but by making inaction visible and timestamped, it creates the conditions under which they can be contested. The CPOC Anemia Guideline (2025) states that ‘PBM should start at the time surgery is booked.^7^ Pillar Zero converts this principle into an institutional rule with a defined patient population and a defined activation event (Table 1). When criteria are met, three actions are triggered simultaneously: the FBC is requested at surgical indication ‒ not at the preoperative assessment; if anemia is identified, structured etiological workup is initiated in parallel with surgical planning; and a documented PBM plan is generated covering the full perioperative period.

**Table 1:** Pillar Zero activation criteria and downstream actions (derived from NICE NG45).^12^

Activation criterion	ASA grade	Action triggered
Major or complex surgery	Any (I–IV)	FBC at surgical indication + PBM pathway open; ownership assigned to surgical team
Intermediate surgery	ASA III or IV only	FBC at surgical indication + PBM pathway open; ownership assigned to surgical team
Minor surgery OR intermediate with ASA I–II	Any	Pillar Zero not activated; standard pathway

ASA, American Society of Anesthesiologists physical status; FBC, Full Blood Count; PBM, Patient Blood Management.

The PBM plan initiated at surgical indication is subsequently reviewed and ratified by an expert anesthetist at the early preoperative assessment ‒ the second governance node of the pathway ‒ who integrates findings into the comprehensive anesthetic risk assessment and coordinates specialist referrals as needed.

No new test is required. The FBC is already indicated by NICE NG45 for this population; the change is only when it is ordered, and by whom. The marginal cost of this displacement is zero. The gain is the 4‒12 week window between surgical indication and surgical date, currently available in most elective settings and systematically wasted.

### The Brazilian context: an unused optimization window and the patient within it

In the Brazilian public health system (SUS), the surgical waiting list ‒ generally viewed as a bottleneck ‒ functions as an optimization window that goes unused.^5^^,^^6^ The 26.7-day median hemoglobin-to-surgery interval confirms that a therapeutic window already exists ‒ sufficient for intravenous iron, and extendable to oral iron when activation occurs at surgical indication rather than at the preoperative assessment; what is missing is not time, but the activation signal. Early risk stratification, described by Grocott as “patient staging” in parallel to pathology staging, creates conditions for shared decision-making, prehabilitation, and comorbidity management that the traditional pathway forecloses.^9^

In the Immich et al. cohort, moderate-to-high functional capacity was the only modifiable variable independently associated with reduced risk of prolonged hospitalization (RR = 0.6),^6^ positioning prehabilitation as a core PBM component equally dependent on early temporal activation.

Screening must adopt a sex-unified hemoglobin threshold of 13 g.dL^-1^ for all adult patients, consistent with the International Consensus Statement^13^ and the 2024 WHO PBM guidance.^14^ Women constituted 82.4% of the mild anemia group in the Immich et al. cohort^6^ ‒ a proportion systematically under detected under sex-differentiated cutoffs. Adopting the unified threshold is a matter of equitable care, one of the IOM’s six foundational quality aims.^8^

The Western Australia statewide PBM program reduced hospital mortality by 28%, transfusion rates by 41%, and generated cost savings of US$ 78–97 million over six years.^15^ In the SUS, Pillar Zero must be incorporated as an institutional performance indicator ‒ a contractual or accreditation metric ‒ rather than a clinical recommendation. Recommendations are filed; indicators are measured, reported, and acted upon.

## Conceptual contribution to the PBM model

The three established pillars are domains of intervention. Pillar Zero is distinct in kind: it is the enabling condition for the other three and the point at which the patient enters the pathway as an active participant rather than a passive recipient. Without it, each pillar remains individually valid, but the three together cannot deliver what they promise, because their shared precondition ‒ time, and patient engagement within that time ‒ is not built into the model itself. Table 2 contrasts the structural consequences of operating with and without Pillar Zero. Pillar Zero is necessary but not sufficient; cultural inertia and fragmented institutional responsibility will continue to attenuate downstream implementation.^4^

**Table 2:** Structural comparison of PBM workflow with and without Pillar Zero, with ESAIC 2025 alignment.^11^

PBM element	Without Pillar Zero	With Pillar Zero	ESAIC 2025 alignment ref.^11^
FBC timing	Preoperative assessment (days before surgery)	Surgical indication (weeks to months before surgery)	R1.4 (CPS): early assessment for high-risk patients to allow optimization
Anemia detected	Too late for oral iron; IV iron often infeasible	Within therapeutic window: oral iron (≥ 4 wk) or IV iron (≥ 2 wk)	R1.3 (CPS): assessment updated within 48h if optimization (e.g., anemia) required
Sex threshold applied	Sex-differentiated cutoffs; women systematically underdetected	Unified Hb < 13 g.dL^-1^ for all adult patients	‒
Patient role	Passive recipient: pathway decided by providers	Active participant in shared decision-making and prehabilitation	R1.2 (1B): telemedicine and standardized questionnaires to improve patient accessibility and satisfaction
Functional assessment	Not assessed in PBM window	Validated functional capacity assessed (DASI or equivalent); prehabilitation initiated if indicated	R3.3 (1A): subjective METs discouraged as standalone measure; DASI recommended
Clinical governance	No named specialist owns the finding	Surgical team activates; anesthetist reviews and ratifies at early preoperative assessment	R2.2 (CPS): expert anesthetist coordinates preoperative evaluation and MDT
Decision available	Proceed with anemia OR cancel surgery	Treat anemia before surgery; proceed optimized	R1.1 (1C): early outpatient assessment reduces day-of-surgery cancellations
Audit metric	Was anemia treated?	Was PBM activated in time ‒ and with the patient ‒ to treat anemia?	‒

DASI, Duke Activity Status Index; FBC, Full Blood Count; IV, Intravenous; MDT, Multidisciplinary Team; PBM, Patient Blood Management; CPS, Clinical Practice Statement; Hb: hemoglobin; METs: Metabolic Equivalents.

A program that audits pillar adherence without auditing activation timing will consistently find protocols followed but outcomes unchanged, because the measurement frame excludes the causal variable. The ICCAMS recommendations^13^ and the Frankfurt Consensus^1^ both recognize early detection as foundational to effective PBM. Pillar Zero formalizes this into an explicit, auditable system requirement: not just ‘was anemia treated?’ but ‘was PBM activated in time ‒ and with the patient ‒ for treatment to be effective?’

## Implementation and digital integration

Pillar Zero’s operational rule ‒ surgical complexity plus ASA classification triggering early FBC ‒ is fully computable. Both variables are already documented at surgical indication, requiring no additional clinical judgment. This makes Pillar Zero uniquely suitable for digital perioperative screening workflows and AI-assisted preoperative assessment tools, where patient-facing interfaces can simultaneously activate hemoglobin screening, functional capacity assessment, and patient education at the moment of surgical indication ‒ consistent with current guideline support for telemedicine and standardized questionnaires as components of preoperative anesthesia care.^11^

In a digital model, when criteria are met the system automatically triggers a physician alert and a draft FBC request; the order is only placed upon explicit physician confirmation, consistent with Brazilian SaMD regulation and the principle that laboratory ordering remains a medical act; upon confirmation, the structured PBM pathway opens with sex-unified thresholds as standard. The activation event must be designed as irreversible within the workflow: non-activation requires explicit documentation, making inaction as visible and accountable as action.

## Conclusion

Patient Blood Management will remain under implemented as long as its temporal precondition goes unnamed, and as long as perioperative pathways are designed around provider convenience rather than patient needs. The evidence is unambiguous: preoperative anemia is prevalent, consequential, and treatable, including in the low-complexity elective procedures that constitute the bulk of any surgical system. The gap between knowledge and action persists because the current model does not specify when action must begin, nor that the patient has a legitimate interest in that decision. Adopting Pillar Zero requires no additional resources, tests, or protocols: only a change in timing and orientation. We call for Pillar Zero to be formally incorporated into the Brazilian PBM framework and implemented in digital perioperative tools as a mandatory activation rule, with sex-unified thresholds, validated functional capacity assessment, and patient engagement as standard components. Patient blood management must begin at the moment surgery is considered, not in the final days before it is performed.

## Data availability statement

No new data were created or analyzed in this study. Data sharing is not applicable to this article.

## Use of AI-assisted technology in writing

AI-assisted tools (Claude, Anthropic) were used to support manuscript drafting and language editing. All scientific content, clinical reasoning, and conclusions were generated and verified by the authors, who assume full responsibility for the integrity of the work.

## Ethics statement

This editorial does not involve human subjects, animal experiments, or patient data. Institutional ethical approval is not required.

## Authors’ contributions (ICMJE)

Flávio Takaoka: Conceptualization; Original draft preparation; Scientific validation; Clinical reasoning; Critical revision for intellectual content, and final approval.

Maria José Carvalho Carmona: Critical revision for important intellectual content; Editorial review, and final approval.

Renato Carneiro de Freitas Chaves: Critical revision for important intellectual content; Co-authorship contributions, and final approval.

Paulo Roberto Silva Garcez dos Santos: Critical revision for important intellectual content; Co-authorship contributions, and final approval.

## Funding

This research did not receive any specific grant from funding agencies in the public, commercial, or not-for-profit sectors.

## Conflicts of interest

The authors declare no conflicts of interest.
